# Deprivation of arginine by recombinant human arginase in prostate cancer cells

**DOI:** 10.1186/1756-8722-5-17

**Published:** 2012-04-30

**Authors:** Eddy C Hsueh, Stephanie M Knebel, Wai-Hung Lo, Yun-Chung Leung, Paul Ning-Man Cheng, Chung-Tsen Hsueh

**Affiliations:** 1 Department of Surgery, Saint Louis University, St. Louis, MO, USA; 2 Department of Applied Biology and Chemical Technology and Lo Ka Chung Centre for Natural Anti-Cancer Drug Development, The Hong Kong Polytechnic University, Hung Hom, Kowloon, Hong Kong, China; 3 Bio-Cancer Treatments International Ltd., Hong Kong Science Park, Bio-informatics Building, Hong Kong, China; 4 Division of Medical Oncology and Hematology, Loma Linda University, Loma Linda, CA, 92354, USA

**Keywords:** Arginase, Prostate cancer, Ornithine carbamoyl transferase, Argininosuccinate synthetase, Mammalian target of rapamycin, Autophagy

## Abstract

**Background:**

Recombinant human arginase (rhArg) has been developed for arginine deprivation therapy in cancer, and is currently under clinical investigation. During pre-clinical evaluation, rhArg has exhibited significant anti-proliferative activity in cancer cells deficient in the expression of ornithine carbamoyl transferase (OCT). Interestingly, a variety of cancer cells such as melanoma and prostate cancer deficient in argininosuccinate synthetase (ASS) are sensitive to arginine deprivation by arginine deiminase. In this study, we investigated levels of gene expression of OCT and ASS, and the effects of rhArg in human prostate cancer cells: LNCaP (androgen-dependent), PC-3 and DU-145 (both androgen-independent).

**Results:**

Quantitative real-time PCR showed minimal to absent gene expression of OCT, but ample expression of ASS expression in all 3 cell lines. Cell viability assay after 72-h exposure of rhArg showed all 3 lines had half maximal inhibitory concentration less than or equal to 0.02 U/ml. Addition of ornithine to cell culture media failed to rescue these cells from rhArg-mediated cytotoxicity.

Decreased phosphorylation of 4E-BP1, a downstream effector of mammalian target of rapamycin (mTOR), was noted in DU-145 and PC-3 after exposure to rhArg. Moreover, there was no significant apoptosis induction after arginine deprivation by rhArg in all 3 prostate cancer cell lines.

**Conclusion:**

rhArg causes significant cytotoxicity in LNCaP, DU-145 and PC-3 prostate cancer cells which all demonstrate decreased OCT expression. Inhibition of mTOR manifested by hypophosphorylation of 4E-BP1 suggests autophagy is involved as alternative cell death mechanism. rhArg demonstrates a promising novel agent for prostate cancer treatment.

## Background

Arginine, a nonessential amino acid, is involved in many biochemical processes besides protein synthesis, such as urea cycle and biosyntheses of creatine, polyamine and nitric oxide [1]. In human tissue, arginine is obtained via protein degradation and dietary intake. Additionally, normal cells can synthesize arginine intracellularly from ornithine, mediated by ornithine carbamoyl transferase (OCT) which metabolizes ornithine and carbamoly phosphate into citrulline; argininosuccinate synthetase (ASS) and argininosuccinate lyase subsequently convert citrulline to arginine [2]. OCT is highly expressed in liver and small intestine, and catabolizes the conversion of ornithine to citrulline [3]. However, OCT expression in cancer and other normal tissues is mostly down-regulated due to epigenetic changes such as DNA hypermethylation [4].

For years, depletion of arginine has been shown to be an effective and promising anti-cancer treatment *in vitro* and *in vivo*[5,6]. By culturing cells in the media depleted of arginine, a variety of human cancer cells have been found to be auxotrophic for arginine, depletion of which results in cell death [7-9]. Further studies have indicated deficiencies in either ASS or OCT expression contributes to arginine auxotrophy in melanoma and hepatocellular carcinoma [10-13]. Arginine can be degraded by three enzymes: arginase, arginine decarboxylase and arginine deiminase (ADI). Both arginine decarboxylase and ADI are not expressed in mammalian cells [2,14]. ADI, an enzyme isolated from *Mycoplasma*, catabolizes arginine to citrulline and ammonia. Pegylated ADI (ADI-PEG20), significantly reduces antigenicity of ADI, and has been evaluated clinically in patients with advanced hepatocellular carcinoma and melanoma [15,16]. The sensitivity of ADI-PEG20 in cancer seems to correspond with deficient expression of ASS. Resistance to ADI-PEG20 has been identified in hepatocellular carcinoma, melanoma and prostate cancer cells expressing ASS [10,17,18].

Arginase participates in the urea cycle, and catabolizes arginine to ornithine and urea [19]. Recombinant human arginase (rhArg) has been developed for arginine deprivation therapy in cancer, and demonstrated significant cytotoxicity in hepatocellular carcinoma and melanoma, *in vitro* and *in vivo*[11-13]. In the setting of OCT deficiency, rhArg eliminates extracellular arginine and results in depletion of intracellular arginine; however in cells expressing OCT, intermediate metabolite such as ornithine can be converted to arginine to avoid intracellular depletion of arginine [20]. It has been demonstrated that OCT deficiency in hepatocellular cancer and melanoma contributes to their sensitivity of growth inhibition by rhArg [11,13]. In contrast to ADI-PEG20, the sensitivity to rhArg in hepatocellular carcinoma and melanoma is independent of ASS expression. Here, we studied the gene expression profile of OCT and ASS, and investigated the effects of rhArg in prostate cancer cells.

## Results and discussion

### Expression of OCT and ASS

Quantitative real-time PCR was performed in prostate cancer cells to detect mRNA expression of ASS, OCT, and glyceraldehyde 3-phosphate dehydrogenase (GAPDH). Data were processed and presented with cycle threshold (Ct) value of each quantitated expression as listed in Table 1. Housekeeping gene GAPDH was used as a reference gene for quantitative expression analysis. The Ct is defined as the number of cycles required for the fluorescent signal to cross the threshold level. Ct is a relative measure of the target mRNA in the PCR, and inversely proportional to the amount of target mRNA. Ct value of 40 or higher means no amplification due to absent gene expression. Abundant expression of ASS mRNA was detected in all three cell lines. Expression of OCT mRNA was absent in LNCaP, and minimally detected in DU-145 and PC-3.

**Table 1:** Cycle threshold (Ct) of argininosuccinate synthetase (ASS), ornithine carbamoyl transferase (OCT), and glyceraldehyde 3-phosphate dehydrogenase (GAPDH) from quantitative real-time PCR in LNCaP, PC-3, and DU-145 cells

	LNCaP	PC-3	DU-145
ASS C_t_	21.07	26.20	22.77
OCT C_t_	40.00	36.82	39.01
GAPDH C_t_	16.53	17.21	17.75

### Cell viability after arginine deprivation by rhArg

**Figure 1:**
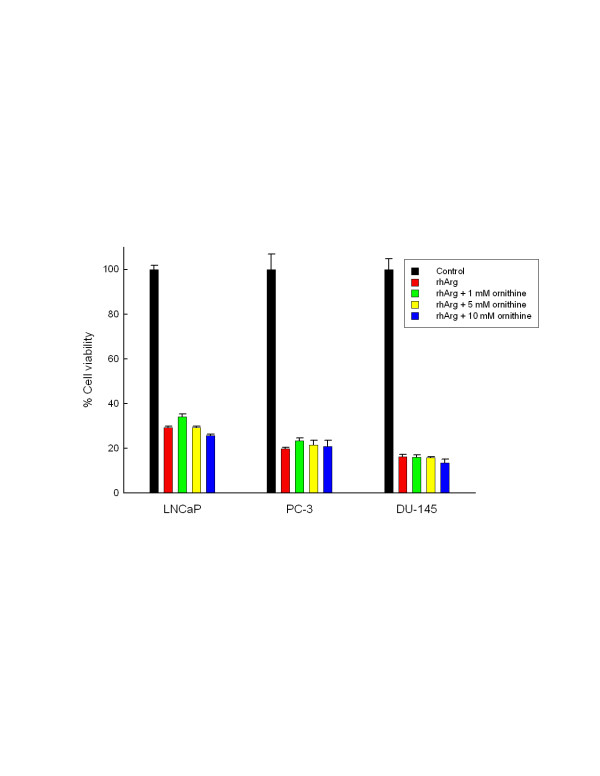
**Cell viability after exposure to rhArg and various concentrations of orinithine.** LNCaP, PC-3 and DU-145 cells were exposed to PBS (control), rhArg (0.1 U/ml), rhArg (0.1 U/ml) + 1 mM ornithine, rhArg (0.1 U/ml) + 5 mM ornithine, and rhArg (0.1 U/ml) + 10 mM ornithine for 72 h, then subjected to cell viability assay as mentioned in Methods. Values were reported as the average of 3 experiments with error bars showing standard error of the mean.

We further determined cell viability after 72-h exposure to rhArg at 0, 0.001, 0.01, 0.1 and 0.5 U/ml. All 3 prostate cancer cell lines were very sensitive to arginine depletion with half maximal inhibitory concentration (IC_50_) of rhArg less than or equal to 0.02 U/ml. The IC_50_ of rhArg in these 3 prostate cancer cell lines was similar to the reported values in melanoma and hepatoma cell lines lacking OCT activity [11,13]. We further investigated the function of OCT by incubating cells with 0.1 U/ml of rhArg (>IC_50_) in the absence and presence of increasing concentrations of ornithine for 72 h. We expected ornithine could rescue rhArg-mediated cytotoxicity if OCT was expressed and functional. As shown in Figure 1, supplement of ornithine failed to rescue cytotoxicity from rhArg in all 3 cell lines. These results were in consistent with deficient OCT gene expression as demonstrated by quantitative real-time PCR.

### Signaling of mammalian target of rapamycin (mTOR)

**Figure 2:**
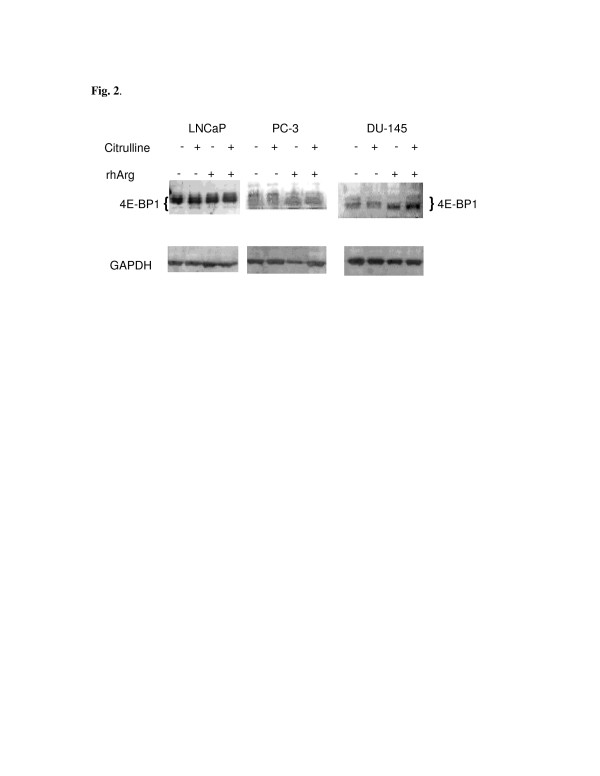
**Western blot analysis of 4E-BP1 and GAPDH (as internal control) in the presence of citrulline, rhArg, or both for 48 h.** Hyperphosphorylated (slowly-migrating with higher molecular weight) and hypophosphorylated (fast-migrating) 4E-BP1 proteins were detected.

Autophagy is mediated by lysosomal degradation, and is an alternative process of cell death [21]. Autophagy is induced by inhibition of mTOR, which is a key sensor and regulator of growth signal and environmental stress [22]. Deprivation of arginine inhibits mTOR pathway and dephosphorylates downstream targets such as 4E-BP1 in Chinese hamster ovary and human melanoma cells [17,23]. To elucidate the mTOR signaling following arginine depletion by rhArg, we investigated the phosphorylation pattern of 4E-BP1. Since our preliminary result showed citrulline could partially reverse the cytotoxicity from rhArg, cells were treated with either citrulline, rhArg, or both for 48 h, followed by Western blot analysis (Figure 2). Decreased phosphorylation of 4E-BP1 in PC-3 and DU-145 was noted upon exposure to rhArg, irrespective of citrulline supplement. However, phosphorylation pattern of 4E-BP1 did not change in LNCaP. Though inhibition of mTOR by rhArg was noted in PC-3 and DU-145, the partial reversal of cytoxicity by citrulline might not be related to mTOR signaling since we did not observe any difference in phosphorylation pattern of 4E-BP1 in the presence or absence of citrulline.

### Measurement of apoptosis

**Figure 3:**
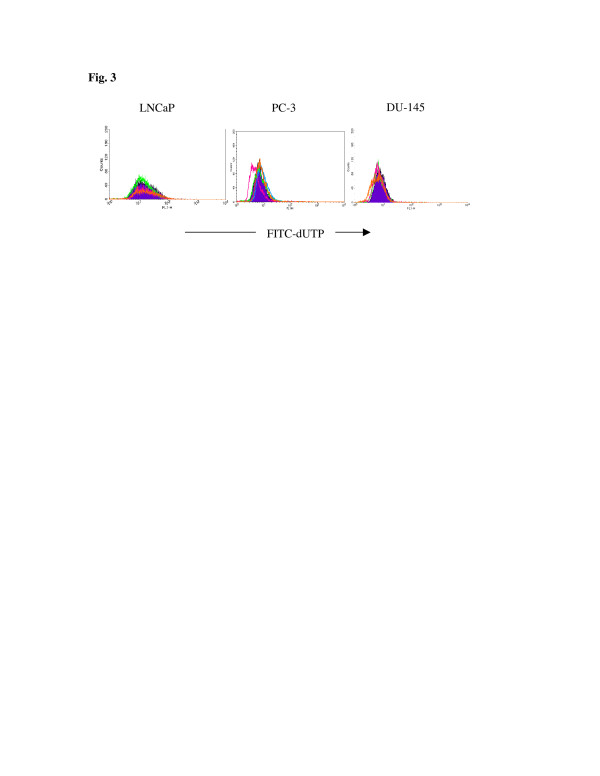
**Determination of apoptosis by TUNEL assay.** Cells were incubated with increasing concentrations of rhArg for 36 h before TUNEL assay and subsequent analysis by flow cytometer. Purple, green, pink, blue, and orange histograms indicate levels of DNA fragmentation with 0, 0.001, 0.01, 0.1, and 0.5 U/ml rhArg, respectively. Y-axis of histogram represents number of cells analyzed, and x-axis represents function of fluorescence intensity resulted from binding of FITC-dUTP (fluorescein isothiocyanate tagged deoxyuridine triphosphate) to fragmented DNA.

Apoptosis was determined by DNA fragmentation using TUNEL (terminal deoxynucleotide transferase dUTP nick end labeling) assay after 36-h co-culture of prostate cancer cells with rhArg. Purple, green, pink, blue, and orange histograms indicate levels of DNA fragmentation upon exposure with 0, 0.001, 0.01, 0.1, and 0.5 U/ml rhArg, respectively (Figure 3). The results demonstrated no induction of apoptosis after 36-h exposure of rhArg.

Deficiency in ASS expression renders cellular sensitivity against ADI-PEG20 in prostate cancer [18]. Both LNCaP and PC-3 have been shown to express ASS, and are resistant to arginine depletion by ADI-PEG20 [18]. In our study, all 3 prostate cancer cell lines including LNCaP and PC-3 expressed ASS but had either minimal or absent expression of OCT, and all 3 lines were highly susceptible to arginine deprivation by rhArg. Moreover, sensitivity to rhArg treatment was independent of hormone sensitivity and not affected by ASS expression in our study.

In human melanoma and prostate cancer cells with down-regulated ASS expression, treatment of ADI-PEG20 activates adenosine 5′-monophosphate-activated protein kinase (AMPK) due to decreased ATP levels upon arginine deprivation [18,24]. Activated AMPK further inhibits mTOR signaling by reducing phosphorylation of 4E-BP1, and leads to autophagy which is a cellular self-degrading process mediated by lysosomes. Kim *et al.* have shown arginine deprivation by ADI-PEG20 immediately activated AMPK, and formed intense autophagosome in CWR22Rv1 prostate cancer cells within 90 min of ADI-PEG20 exposure [18]. Onset of caspase-independent apoptosis in ~30% CWR22Rv1 cells did not occur until after 96-h exposure of ADI-PEG20. Similar findings of delayed-onset but caspase-dependent apoptosis after arginine deprivation with 3 to 6 days exposure of either ADI-PEG20 or rhArg were reported by different groups [13,24].

Common stimuli can induce autophagy and apoptosis, which occur either in combined manner or sequential event [25]. It is unclear about the functional relationship between autophagy and apoptosis upon arginine deprivation with either ADI-PEG20 or rhArg. It is possible that upon initial arginine deprivation, autophagy is activated as a defense mechanism to suppress caspase-dependent apoptosis. As arginine deprivation persists more than 72 h, autophagy may give in to caspase-dependent apoptosis in some cell types; whereas in certain cancer cells, autophagy lasting longer than 24 h may lead to caspase-independent form of programmed cell death (autophagic type II cell death) [26].

Using culture media deficient in L-arginine, Wheatley *et al.* studied the effects of arginine deprivation in 26 cancer cell lines, including PC-3 [27]. They demonstrated clear evidence of cell death during second day of arginine deprivation, and most of PC-3 cells died within 3 days. Furthermore, they observed significantly increased phagosome/lysosome activity from 24 to 36 h after arginine deprivation, suggestive of lytic cell death such as autophagy rather than apoptosis. In this study, we did not identify any significant apoptosis induction after 36-h exposure of rhArg in all 3 cell lines. Additionally, inhibition of mTOR signaling manifested by decreased phosphorylation of 4E-BP1 was noted in DU-145 and PC-3 cells after 48-h exposure of rhArg. Our results are consistent with the report from Wheatley and others, indicating cell death by arginine deprivation in DU-145 and PC-3 is due to autophagic cell death.

Both rhArg and ADI are developed for arginine deprivation in cancer treatment, and currently undergoing clinical investigation. rhArg exhibits significant cytotoxicity against cancer cells such as prostate cancer, melanoma, and hepatocellular carcinoma with OCT deficiency. ADI is effective in tumor cells lacking ASS. Therefore, cancer can be ADI-resistant but rhArg-sensitive, and vice versa. Personalized medicine can be achieved by examining the expression of OCT and ASS in cancer specimen before subjecting cancer patients to arginine deprivation therapy.

## Conclusions

rhArg causes significant cytotoxicity in LNCaP, DU-145 and PC-3 prostate cancer cells. The cytotoxicity of rhArg correlates with deficient OCT gene expression, but is independent of hormone sensitivity and not affected by ASS gene expression. Inhibition of mTOR signaling, manifested by reduced phosphorylation of 4E-BP1, suggests autophagy is involved as alternative cell death mechanism. rhArg is a promising targeted agent for prostate cancer, and its activity and mechanism of action warrant further validation by clinical investigation.

## Methods

### Cell culture

DU-145, LNCaP and PC-3 human prostate cancer cells were obtained from the American Type Culture Collection (Manassas, VA). DU-145 and PC-3 are androgen-independent, and LNCaP is androgen-dependent [28]. Cell lines were maintained in RPMI 1640 medium (Life Technologies, Grand Island, NY) supplemented with 10% fetal bovine serum and antibiotics at 37°C in a humidified atmosphere of 5% CO_2_. rhArg was kindly provided by Bio-Cancer Treatments International Ltd. (Hong Kong, China), and was characterized as described previously [11].

### Quantitative real-time PCR

Total RNA was extracted using TRIzol reagent (Life Technologies), and cDNA was transcribed from total RNA using SuperScript II RT kit (Life Technologies). Quantitative real-time PCR was performed in triplicate on a 7300 Real Time PCR System, using Gene Expression Assays for ASS, OCT, and GAPDH genes (Applied Biosystems, Foster City, CA). Data were processed and presented with Ct value of each gene expression.

### Cell viability assay

Cells were plated at 10^4^ cells per well in a 96-well plate with increasing concentrations of rhArg at 0, 0.001, 0.01, 0.1 and 0.5 U/ml for 72 h at 37^0^ C. Subsequently, cell viability was determined by a colorimetric method using CellTiter 96 Aqueous Non-radioactive Cell Proliferation Assay according to the manufacturer’s protocol (Promega, Madison,WI).

### Protein extraction and Western blot analysis

Protein extraction and Western blot analysis were carried out as previously described with some modifications [29]. After treatment, cells were washed twice with cold phosphate-buffered saline, and then resuspended in lysis buffer (phosphate-buffered saline containing 1% Nonidet P-40, 0.5% sodium deoxycholate and 0.1% SDS) containing the protease inhibitors (100 μg/ml phenylmethylsulfonyl fluoride, 25 μg/ml aprotinin, 25 μg/ml leupeptin, 10 μg/ml soybean trypsin inhibitor and 1 mM sodium orthovanadate). The lysate was incubated on ice for 30 min, passed through a 21 gauge needle twice, and then centrifuged at 15,000 x g for 20 min at 4°C. Protein concentration was determined using the Bio-Rad protein assay. Whole cell lysate containing 50 μg of protein from each sample were used in immunoblotting, and subsequently the gels were electroblotted onto PVDF membranes (Immobilon-P, Millipore). Antibodies purchased from Cell Signaling Technology (Danvers, MA) were used to detect the proteins of interest. The horseradish peroxidase conjugated antibodies against mouse, rabbit and goat IgG were used as secondary antibodies (Sigma-Aldrich, St. Louis, MO). The secondary antibody binding was detected by ECL Plus chemiluminescent reagents and analyzed by Storm image analysis systems (GE Healthcare Biosciences, Piscataway, NJ).

### Apoptosis

Apoptosis was determined by DNA fragmentation using ApoDirect TUNEL assay kit from Millipore (Billerica, MA) based on supplier’s instruction. Briefly, 10^6^ Cells were incubated with increasing concentrations of rhArg for 36 h. Afterwards, DNA breaks were fluorescently labeled with fluorescein isothiocyanate, and cells were analyzed by FACScan flow cytometer (Becton Dickinson Biosciences, San Jose, CA) using Cell Quest Pro software.

### Statistical analysis

All experiments have been performed at least twice with similar results, and the results of one representative experiment are reported. Cell viability results are reported as the average of 3 experiments with error bars representing standard error of the mean as shown in Figure 1.

## Abbreviations

OCT = Ornithine carbamoyl transferase; ASS = Argininosuccinate synthetase; ADI = Arginine deiminase; ADI-PEG20 = Pegylated ADI; rhArg = Recombinant human arginase; GAPDH = Glyceraldehyde 3-phosphate dehydrogenase; Ct = Cycle threshold; IC50 = Half maximal inhibitory concentration; mTOR = Mammalian target of rapamycin; TUNEL = Terminal deoxynucleotide transferase dUTP nick end labeling; AMPK = Adenosine 5′-monophosphate-activated protein kinase.

## Competing interests

PNMC is the chief executive officer of Bio-Cancer Treatments International Ltd, which manufactures rhArg. The rest of the authors declare no competing interest.

## Authors’ contributions

ECH, WHL, YCL, PNMC and CTH designed the experiments. ECH and SMK performed the experiments. ECH and CTH wrote the paper. All authors read and approved the final manuscript.
